# Managed Entry Agreements for Pharmaceutical Products in Three Maghreb Countries: Payer and Supplier Perspectives

**DOI:** 10.3390/jmahp13030040

**Published:** 2025-08-11

**Authors:** Hajer Dahmani, Leila Achour, Maghreb Research Group, Mondher Toumi, Ines Fradi

**Affiliations:** 1 Department of Pharmaceutical Quality Assessment, National Laboratory of Control of Medicines, Tunis 1006, Tunisia; 2 Faculty of Pharmacy of Monastir, University of Monastir, Monastir 5000, Tunisia; leila.achourr@gmail.com (L.A.);; 3 Laboratory LR18ES39, University of Tunis Manar, Tunis 1068, Tunisia; 4 CEReSS/UR3279—Health Services Research and Quality of Life Center, Aix-Marseille University, 13385 Marseille, France; 5 InovIntell, 215 rue du Faubourg St Honoré, 75008 Paris, France

**Keywords:** health economics, managed entry agreements, Tunisia, Morocco, Algeria

## Abstract

Our objective is to describe the experience and challenges of using Managed Entry Agreements (MEAs) in Algeria, Morocco, and Tunisia. We conducted online interviews with key decision-makers in Algeria, Morocco, and Tunisia between March 2021 and December 2023. The questionnaire captured experience with MEAs, types of agreements implemented, and challenges to implementing MEAs. Three, five, and seven participants working, respectively, in the Algerian, Moroccan, and Tunisian pharmaceutical sectors, participated in the interviews. Participants were from the public (8/15) and the private sector (7/15). Only Tunisian respondents reported having dealt with MEAs contracts, such as financial-based agreements (FBAs) related to standard discounts and volume-based price discounts. All respondents were aware of the potential need for structuring contracts differently for expensive medicines. Hurdles in implementing MEAs noted by respondents were mainly related to the absence of a legal framework and the lack of budget allocated for new medicines. Most respondents projected an increase in the use of MEAs to improve reimbursement and access to new, highly priced medicines. Recommendations include strengthening pricing, reimbursement processes, and HTA use. The adoption of FBAs is suggested as a practical initial approach.

## 1. Introduction

An overarching goal for health authorities all over the world is to provide timely access to new medicines and technologies that are safe and effective. New medicines often come with high prices and limited evidence of therapeutic benefit at the time of launch [1], creating significant challenges for payers due to budget constraints [2,3]. To address this issue, payers and the pharmaceutical industry have increasingly adopted Managed Entry Agreements (MEAs), which have become common in many European countries [4,5,6,7,8,9,10,11,12,13]. The World Health Organization, in collaboration with the Organization for Economic Co-operation and Development (OECD), has defined MEAs as “arrangements between a manufacturer and payer/provider that enable access to (coverage/reimbursement of) a health technology subject to specified conditions. These arrangements can use a variety of mechanisms to address uncertainty about the performance of technologies or to manage the adoption of technologies in order to maximize their effective use, or limit their budget impact” [14,15]. In general, there are two main types of MEAs: financial-based agreements (FBAs) and performance-based agreements (PBAs). Financial mechanisms aim to target the financial impact of new medicines on patients and/or health systems and rely on instruments such as rebates, price/volume agreements, patient/dose-based rebates, and utilization-based price caps. Conversely, PBAs respond to uncertainty about the evidence on clinical outcomes or eligibility of patient populations [14]. Experience with FBAs is extensive, while experience with PBAs is more limited [16,17]. PBAs were found mostly in the US and UK, but also in Italy, Germany, the Netherlands, and Sweden, because of political encouragement, cultural acceptance, and the existence of decision-making to agree to such a scheme [18]. PBAs were also described in Australia [19]. Other models that combine FBAs and PBAs have been categorized as “hybrid” [20].

Although some information on MEAs exists in the context of European countries, limited information is available about MEAs’ use in Maghreb countries, where pricing and reimbursement processes are mainly based on External Reference Pricing(ERP) [21,22]. In fact, whereas each country has its specific regulations, there are many similarities in the pricing and reimbursement policies of in-patent medicines in Algeria, Morocco, and Tunisia. The ERP was found to be the dominant method to inform pricing and reimbursement decisions of in-patent medicines.

Algeria, Morocco, and Tunisia are North African countries, collectively known as Maghreb countries, each with unique economic conditions and fragmented healthcare systems [21].

This research paper aims to assess the current level of implementation of MEAs in Algeria, Morocco, and Tunisia and to analyze the potential barriers related to their implementation in the Maghreb region. The ultimate goal of this study is to propose recommendations for the implementation of MEAs in the studied countries.

## 2. Materials and Methods

A prospective cross-sectional survey was conducted between March 2021 and December 2023. A cover letter and a Google form questionnaire were sent by email to 25 stakeholders from Algeria, Morocco, and Tunisia. The respondents were selected based on their current positions and their experience in the field. All the respondents were potential decision-makers working in the public and private sectors with solid experience in pricing and reimbursement). They were asked to fill out the predesigned questionnaire that included 9 questions to capture: (i) previous and current experience with MEAs, types of agreements implemented by therapeutics areas; (ii) challenges and barriers to implementing MEAs; and (iii) stakeholders’ perspectives about implementing MEAs by therapeutic area (Table 1). The authors had no contact with the reviewers other than for the study.

The data were collected from the response and then analyzed by themes according to the analytical framework.

This study does not describe the pricing and reimbursement systems in these 3 countries since they were previously outlined in the article published in 2023 [21].

## 3. Results

A total of 15 participants (60%) responded to the survey and interview requests, with 8 (53%) coming from the public sector. Most respondents held important positions in pricing and reimbursement decision-making (Table 2).

### 3.1. MEAs’ Perceptionsin Tunisia, Algeria, and Morocco and Stakeholders’ Perspectives

Most respondents expressed a positive perception of the future of MEAs in the region. These contracts are becoming very important for payers due to pressures on healthcare budgets.

From the manufacturers’ perspective, negotiating confidential price agreements is crucial to avoid impacting prices in other countries, particularly in those where the practice of ERP is the main pricing method.

Some respondents, mainly from the public sector, reported a lack of awareness regarding the rationale behind each type of contract. They noted a lack of clarity regarding the rationale behind choosing a specific type of contract for particular therapies, such as treatments for orphan diseases or gene and cell therapies. PBAs seem to be the best choice for oncology treatments (Morocco and Tunisia), as they would allow for better savings. However, some respondents were familiar with FBAs, as these contracts are suitable for products whose costs cannot be borne by payers and whose patient numbers are predictable. Financial contracts seem to be easier to understand and implement than outcome-based schemes, and can help contain costs and keep expenditure within an agreed limit.

Regarding the therapeutic area of interest for MEAs in the region, the survey findings indicate that these agreements are likely to primarily involve oncology and immune-modulating products. This is consistent with the European trend and supports payers’ rationale for entering MEAs for costly medicines [23]. Orphan drugs, being very expensive treatments, are also considered candidates for MEAs. In Algeria, interviewees reported that MEAs would be interesting for hemophilia treatments because the Algerian patients’ association of hemophilia is actively working to improve access to treatment. Additionally, Algerian respondents suggested that MEAs could be applied to drugs for autoimmune diseases, such as Elaprase, Imiglucerase, and Velaglucerase.

### 3.2. MEAs Experience in Algeria, Morocco, and Tunisia

The general information about MAEs’ experience is summarized in Table 3.

#### 3.2.1. MEAs Experience in Tunisia

Access to new medicines in Tunisia is still challenging, mainly due to high prices.

The National Health Insurance (CNAM) remains the principal option for patients to obtain their new high-cost therapies. If CNAM refuses to cover the costs, patients turn to the courts to assert their right to health. Consequently, the CNAM is compelled to cover treatments from suppliers at exorbitant prices since this treatment is intended for a single person. For patients with no health insurance coverage, the Ministry of Health is attempting to address access issues by initiating an agreement with the pharmaceutical industry called the “Patient Access Program”. This program includes in the price a condition in the price negotiation that requires the provision of a percentage of free goods intended for these patients.

This type of contract has facilitated the implementation of MEAs in Tunisia. Since 2023, two FBPs have been performed for reimbursement of advanced therapies in oncology. These FBAs mostly involve standard discounts and volume agreements between the manufacturer, the payer (CNAM), and the purchaser (PCT). However, there are no published details.

Nevertheless, respondents reported several significant barriers that slow down the implementation of these contracts in Tunisia, including:

- Legal texts that are not aligned with current health system developments.
- The complexity of the pricing and reimbursement system.
- Lengthy timelines for granting marketing authorization.
- A lack of alignment between resource allocation and the budget impact of new therapies that should be selected to meet health needs and priorities.

To improve patients’ access to new medicines, especially expensive drugs, respondents recommend:

- Revising the pricing and reimbursement process.
- Including cost-effectiveness evaluations from the HTA body (INEAs) to allocate the budget and allow for the reimbursement of new high-cost medicines.
- Creating new negotiation processes, such as a unique committee as a decision-making body for pricing, and utilizing MEAs for reimbursement purposes.

#### 3.2.2. MEAs Experience in Algeria

Similarly to Tunisia, access to new high-cost medicines in Algeria remains challenging due to high prices and a limited healthcare budget. Although the Ministry of Labor and Social Security set provisions about MEAs in the new finance law of 2017, none of the respondents reported that any MEA has been applied in Algeria to date. It seems that the law introduced the principles, but the development of a regulatory framework is still needed to describe how to implement MEAs.

Respondents reported that the absence of a detailed legal framework and the lack of experience with MEAs represent the main barriers to implementing these contracts in Algeria. They noted that FBAs are more appropriate and easier to implement by the National Insurance Scheme as part of the reimbursement process, mainly for cancer therapeutic areas. According to the respondents, the extension of the healthcare budget, for example, to include cancer plans and rare disease plans, would facilitate the implementation of MEAs.

In Algeria, stakeholders did not have the same level of awareness about the MEA’s utility. Although some stakeholders were interested in these contracts, they were somewhat afraid of changing their negotiating methods. A contract called a pay-for-performance agreement with pharmaceutical companies for the reimbursement of new, high-cost medicines has been described in Algeria. It conditions the reimbursement by the performance (medicines will not be reimbursed in case of therapeutic failure) [24]. According to Algerian respondents, MEAs would be more relevant to be implemented in the in-patient sector (hospital market).

#### 3.2.3. MEAs Experience in Morocco

In Morocco, there are difficulties in accessing the market for new, high-cost therapies. This is due to several factors, as reported by the interviewees: (i) affordability issues related to the low rate of health insurance coverage; (ii) the long and uncertain reimbursement process; and (iii) the registration timelines.

Some solutions were undertaken, such as:

- Universal Health Coverage launched in 2021 with a target of 100 of % total populaion by the end of 2022;
- Fast Track registration allocated to products with a high public health interest (subject to evaluation by the Ministry of Health and Social Protection);
- The Lalla Salma Association Against Cancer and local manufacturing of hepatitis C treatment and its marketing at an affordable price for Moroccan patients [25].

MEAs have not yet been experienced in Morocco. According to respondents, MEAs will be introduced in the process of pricing and reimbursement of new, high-cost therapies. It would be mainly FBAs.

### 3.3. Challenges for the Implementation of MEAs in Tunisia, Algeria, and Morocco

Interviewees reported many challenges for the implementation of MEAs in the study countries, such as:

- An absence of legal/policy framework: There is no law that explains the implementation methods for innovative contracts, nor the responsibilities of stakeholders, nor the confidentiality of the contract.
- A lack of an effective information system enabling disease registries and databases: There are no registers to monitor the treatment results.
- The existence of multiple levels for decision-making and the absence of a unique pricing and reimbursement committee in Tunisia or Morocco to optimize negotiation mechanisms.
- The limited size of the market or target population for some types of contracts: For example, orphan diseases, where the nature of the contract for these types of treatments can only be a performance contract. The issue is the absence of registers.
- The lack of budget allocation: Authorities face a lack of budget to implement these contracts, which can be costly, especially performance contracts with dedicated registers, human resources for monitoring and maintenance, as well as data collection and analysis.
- The limited application of health technology assessment (HTA) to enable evidence-based decision-making by the payer (and other stakeholders) and to facilitate MEAs negotiation.
- A lack of experience in terms of the establishment and assessment of contract terms and definitions.
- Manufacturers’ awareness about the confidentiality of discounts and rebates on the MEAs: Private sector actors have expressed concerns about the confidentiality of contracts. Negotiating confidential price agreements is crucial to avoid impacting prices in other countries and harming the business of pharmaceutical companies.

### 3.4. Facilitating Elements to the Implementation of MEAs in the Studied Countries

The facilitating elements to MEA implementation are summarized in Table 4.

#### 3.4.1. Facilitating Elements to the Implementation of MEAs in Tunisia

According to the respondents, some elements facilitated the implementation of MEAs in Tunisia.

The first factor is the early dialog with companies and various institutions, which has been ongoing since 2018 and has increased awareness of MEAs and their potential benefits.

The second factor is the initiation of HTA. Indeed, several reports have been established by INEAS and could be used for the PBAs’ implementation in some therapeutic areas.

The third factor is the role played by the Medicine Purchasing Committee (CAM) at the PCT, which can be the decision-making body in MEA’s assessment and agreement.

Finally, sales data is available in the PCT database, and reimbursement data is available in the CNAM database, which represents an encouraging starting point for the FBAs implementation, in which we need to follow the sales volume and reimbursement amount.

#### 3.4.2. Facilitating Elements to the Implementation of MEAs in Algeria

Respondents did not explicitly report facilitating elements to the implementation of MEAs in Algeria. Nevertheless, the use of budget impact analysis as a pricing policy in Algeria may help in implementing MEAs [21].

#### 3.4.3. Facilitating Elements to the Implementation of MEAs in Morocco

Interviewees claimed that industry commitment could be considered as a facilitating element to the MEAs’ implementation. The willingness of public authorities to improve access of Moroccan patients to new, high-cost medicines also represents an opportunity that could help in accelerating MEA implementation.

## 4. Discussion and Recommendations for the MEAs’ Implementation in Tunisia, Algeria, and Morocco

This study highlights that MEAs are being initiated in Maghreb countries, starting with Tunisia, which established two FBAs in 2023. Additionally, regulatory texts are being developed in Algeria, and there are attempts to implement MEAs in Morocco.

The survey revealed that, from the perspective of the interviewed stakeholders, FBAs would be more easily applicable in the studied countries. This is primarily due to the limited application of HTA and the lack of evidence available to establish thresholds for treatment response in PBAs.

On the other hand, based on the experience of high-income countries (HICs), the administrative burden required for implementation, including the development and maintenance of patient registries, should be considered by payers when deciding whether and how to implement MEAs. Moreover, an observational study of the financial outcomes of MEAs in Italy revealed that, while medicinal products were more frequently subject to PBAs than to FBAs, the majority of paybacks come from FBAs [26]. A meta-analysis based on the literature review showed that 97% of MEAs identified involved medicines, typically medicines for oncology, with FBA as the most prevalent scheme [27].

As a result of this study, some recommendations could be considered for the implementation of MEAs in Tunisia, Algeria, and Morocco to improve the management of pharmaceutical expenditures:

- The Tunisian case showed that the absence of a specific legal framework is not the primary challenge for the effective implementation of MEAS. However, the establishment of a detailed legal framework would provide standardized practices and offer certain assurances to pharmaceutical companies regarding the respect of price confidentiality.
- Strengthening the pricing and reimbursement process in Morocco and Tunisia through a unique committee that includes all healthcare decision-makers under the management of a single organization, facilitating pertinent data entry, analysis, as well as monitoring of any agreements.
- Improvement of the data collection and patients’ registries is crucial for estimating the budget impact and better informing the pricing and reimbursement decision-making.
- Enhancement of the HTA use with a focus on oncology, orphan drugs, and expensive therapies.
- The adoption of FBAs would be more applicable and appropriate in the studied countries.
- Building capacities of stakeholders to enable the effective implementation of MEAs with appropriate design for prioritized medicines based on strong scientific evidence.

## 5. Limitations

This study has some limitations. As this topic is very specific, there is a limited number of healthcare specialists and decision-makers in MEAs in each country, making it difficult to include a large number of participants in this study. Additionally, complete data sets could not be obtained due to limited publications about MEA experience in Maghreb countries and the confidential nature of the MEAs’ contracts. Nevertheless, we believe this study was the first attempt to evaluate North African countries’ experience with MEA contracts.

## 6. Conclusions

This study sheds light on the current state and challenges of implementing MEAs in Algeria, Morocco, and Tunisia. Despite their widespread use in European countries, MEAs are still in the early stages of adoption in Maghreb countries. MEAs may offer significant benefits in these countries, where the introduction of new, high-cost medicines often faces challenges due to limited healthcare budgets. By implementing MEAs, these countries can manage the financial risks associated with high-cost medicines, which encourages quicker access to innovative, high-cost therapies for patients, even in the absence of comprehensive long-term data. Additionally, MEAs enable healthcare systems in the Maghreb region to better control expenditures while maintaining access to treatments. Moreover, these agreements offer an opportunity for ongoing real-world data collection, providing valuable evidence that can refine treatment protocols and pricing strategies in the future. Nevertheless, several barriers to effective implementation exist, including the absence of a legal framework, a lack of effective information systems for disease registries and databases, and multiple levels of decision-making. This study provides several recommendations for the implementation of MEAs in Tunisia, Algeria, and Morocco to better manage pharmaceutical expenditures. Strengthening the pricing and reimbursement processes, improving data collection and patient registries, and enhancing the use of HTA are crucial steps for these countries. Ultimately, the adoption of FBAs could provide a more practical and appropriate approach in the initial stages.

## Figures and Tables

**Table 1:** Analytical framework and key endpoints.

Key Themes of the Analytical Framework	Key Endpoints	The Objectives of the Framework Theme and Associated Endpoints
Current and previous experience with MEAs	Types of MEAs Number of MEAs	Describing stakeholders’ experience with MEAs, types and number of MEAs established, if they exist
Perception of MEAs	Utility of MEAs The rationale behind each type of MEA	Understanding of the perception of stakeholders of each type of MEA and the rationale behind FBAs and PBAs
Hurdles to the implementation of MEAs	Barriers to the implementation of MEAs	Describing the different barriers to the implementation of MEAs
Perspectives on MEAs’ implementation and outlook	Stakeholders’ interest in implementing MEAs Stakeholders who should be involved in the MEAs implementation and monitoring Therapeutic areas that would be potentially affected by MEAs	Understanding stakeholders’ interests and perspectives on the future implementation of MEAs, potential stakeholders involved, and therapeutic areas concerned by these agreements

**Table 2:** Respondents’ demographics.

	Algeria	Morocco	Tunisia
**Number of Participants**			
Public sector	1	2	5
Pharmaceutical industry	2	3	2
**Public Participants occupations**			
Pricing	1	1	1
Health Technology Assessment (HTA)	-	-	1
Reimbursement	-	1	1
Purchasing/sales	-	-	2
**Private Participants occupations**			
Market Access	1	3	2
Sales	1	-	-

**Table 3:** MEAs’ experience in Tunisia, Algeria, and Morocco.

Country	Summary of MEA’s Experience
Tunisia	MEAs are just starting in Tunisia. Two FBAs have been performed since 2023. These contracts involve standard discounts and volume agreements between the manufacturer, the payer (CNAM), and the purchaser (PCT). Based on the quantities purchased, the manufacturer provides an agreed number of free boxes in the form of a credit note to the PCT, which is then passed on to the CNAM. The proportion of goods is confidential.
Algeria	Although the Ministry of Labor and Social Security set provisions about MEAs in the new finance law of 2017, no MEA has been performed yet. Respondents reported that FBAs are more appropriate and easier to implement by the National Insurance Scheme as part of the reimbursement process, mainly for cancer therapeutic areas.
Morocco	MEAs have not been experienced in Morocco yet. According to respondents, MEAs will be introduced in the process of pricing and reimbursement of new high-cost therapies. It would be mainly FBAs (Standard discounts, Price/volume agreement, Capped volume, and Capped budget) for oncology treatments.

**Table 4:** MEAs facilitating elements in Tunisia, Algeria, and Morocco.

Countries	Facilitating Elements to MEA Implementation
Tunisia	The early dialog with companies and various institutions The initiation of HTA The presence of the Medicine Purchasing Committee (CAM) at the PCT can be the decision-making body in MEAs The availability of sales data: sales volume and reimbursement amount
Algeria	The use of budget impact analysis in pricing and reimbursement policy The inclusion of MEAs in the new finance law of 2017
Morocco	The industry commitment

## Data Availability

The original contributions presented in the study are included in the article, further inquiries can be directed to the corresponding authors.
